# Adaptive Wavelet Based MRI Brain Image De-noising

**DOI:** 10.3389/fnins.2020.00728

**Published:** 2020-07-22

**Authors:** Noorbakhsh Amiri Golilarz, Hui Gao, Rajesh Kumar, Liaqat Ali, Yan Fu, Chun Li

**Affiliations:** ^1^School of Computer Science and Engineering, University of Electronic Science and Technology of China, Chengdu, China; ^2^School of Information and Communication Engineering, University of Electronic Science and Technology of China, Chengdu, China; ^3^The 54th Research Institute of China Electronics Technology Group Corporation, Shijiazhuang, China

**Keywords:** wavelet, MRI image de-noising, AGGD, adaptive threshold, PSNR

## Abstract

This paper presents a unique approach for wavelet-based MRI brain image de-noising. Adaptive soft and hard threshold functions are first proposed to improve the results of standard soft and hard threshold functions for image de-noising in the wavelet domain. Then, we applied the newly emerged improved adaptive generalized Gaussian distributed oriented threshold function (improved AGGD) on the MRI images to improve the results of the adaptive soft and hard threshold functions and also to display, this non-linear and data-driven function can work promisingly even in de-noising the medical images. The most important characteristic of this function is that it is dependent on the image since it is combined with an adaptive generalized Gaussian distribution function.Traditional thresholding neural network (TNN) and optimized based noise reduction have good results but fail to keep the visual quality and may blur some parts of an image. In TNN and optimized based image de-noising, it was required to use Least-mean-square (LMS) learning and optimization algorithms, respectively to find the optimum threshold value and parameters of the threshold functions which was time consuming. To address these issues, the improved AGGD based image de-noising approach is introduced to enhance the qualitative and quantitative performance of the above mentioned image de-noising techniques. De-noising using improved AGGD threshold function provides better results in terms of Peak Signal to Noise Ratio (PSNR) and also faster processing time since there is no need to use any Least-mean-square (LMS) learning and optimization algorithms for obtaining the optimum value and parameters of the thresholding functions. The experimental results indicate that image de-noising using improved AGGD threshold performs pretty well comparing with the adaptive threshold, standard threshold, improved wavelet threshold, and the optimized based noise reduction methods.

## 1. Introduction

Image de-noising is among the most important tasks in image and signal processing. Wide range of unwanted noises may affect the visual quality of images. The noise can affect an image during the processes of acquisition and transmission which can cause deflection from an original image. It is obvious that the image quality and resolution may be contaminated by these artifacts so that it is required to do image de-noising as the first step before any further analysis, such as super-resolution, classification and any qualitative and quantitative measurement.

One of the most crucial issues in image de-noising is keeping the most influential characteristics of the images and removing the non-important characteristics. Noise removal has become one of the critical pre-processing steps in many applications like remote sensing, satellite and biomedical image processing (Golilarz et al., 2019b). Some of these noises can affect the appearance and damage the attribute of an image. Others may not be continuous and they occur randomly. In this case, it is very difficult to get rid of these kinds of noises. However, many methods have been proposed for reducing the possible noises from the images and enhancing their quality.

Yuan and Ghanem (2015) introduced a new approach for image restoration in the presence of impulse noise. Weighted couple sparse representation has been introduced by Chen et al. (2015). To remove multimodal noise using semi-supervised learning on big data, Yin et al. (2018) introduced a highly accurate image reconstruction. Garnett et al. (2005) introduced a universal noise reduction algorithm combined with an impulse detector. Median- type noise detectors and detail-preserving regularization based noise removal are proposed by Chan et al. (2005). Moreover, impulse noise reduction with Gaussian curvature of image surface is proposed by Miura et al. (2013). Lin et al. (2010) introduced impulse noise suppression using a new adaptive center weighted median (ACWM) filter. A new impulse detector combined with weighted median filter is proposed by Dong and Xu (2007) to obtain the directional weighted median (DWM) filter. Universal noise reduction using a switching bilateral filter combined with a noise detector is utilized by Lin et al. (2010). The standard deviation to acquire the optimal direction is proposed by Awad (2011) as a new technique to discard the noise from images influenced by random-valued impulse noise. In 2013, Lu et al. proposed sparse coding for noise removing with spike and slab prior (Lu et al., 2013). Noise reduction utilizing a scale mixture of Gaussians was presented by Portilla et al. (2003). In this technique, the components have been explained with a statistical model. Additionally, the estimation of mode in high-dimensional spaces using flat-top kernels has been proposed in a study conducted by De Decker et al. (2011). Recently, wavelet and thresholding based noise reduction has become very common among researchers in the field of image and signal processing. Many techniques have been introduced to discard the noises and keep the most significant characteristics of images in the wavelet domain.

Chang et al. (2000) proposed context modeling for parameter estimation of each component which is adaptive to wavelet thresholding. It is clear, this component is modeled as a random variable for GGD. Based on the obtained results, this method has better performance than orthogonal transform. Speckle noise reduction utilizing a Bayesian multiscale method is introduced by Achim et al. (2001). An empirical Bayes method with Jeffrey's non-informative prior is also a noise removal method based on wavelet transform proposed by Figueiredo and Nowak (2001). Şendur and Selesnick (2002) proposed bivariate shrinkage function for image denoising using wavelet transform. Image de-noising using joint inter- and interscale statistical model, translation invariant wavelet transformations, and Bayesian wavelet shrinkage based on heavy-tailed modeling have been proposed by Pizurica et al. (2002), Achim et al. (2003), and Sveinsson and Benediktsson (2003), respectively.

Starck et al. (2002) proposed the curvelet transform for noise reduction. Additionally, sparse and redundant representations over learned dictionaries for noise removing is proposed by Elad and Aharon (2006). The local adaptive wiener filter approach for noise suppression in wavelet domain is introduced by Li et al. (2011). Image de-noising with an un-decimated wavelet transform (UWT) utilizing soft thresholding function is introduced by Golilarz and Demirel (2018a). Furthermore, image de-noising based on translation invariant wavelet transform combined with smooth sigmoid based shrinkage (SSBS) function is introduced by Golilarz et al. (2017). Adapting to unknown smoothness via wavelet shrinkage is introduced by Donoho and Johnstone (1995).

To improve the quality and performance of the previous method, Coifman and Donoho (1995) proposed translation-invariant de-noising. Numerous literature has emerged for thresholding neural network (TNN) based noise suppression. Thresholding neural network (TNN) for adaptive noise reduction is proposed by Zhang (2001). In this study, new types of soft and hard threshold functions have been presented to be utilized as the activation function in TNN. These threshold functions are differentiable and non-linear. Moreover, thresholding neural network-based noise reduction with a smooth sigmoid based shrinkage and TNN using an improved threshold function have been proposed by Golilarz and Demirel (2017) and Golilarz and Demirel (2018b), respectively. Image denoising in the wavelet domain based on improved TNN and cycle spinning has been conducted in a study proposed by Sahraeian et al. (2007). In this study, the authors utilized a new adaptive improved threshold function combined with cycle spinning to enhance the results of TNN based image de-noising using adaptive thresholding. Besides, Nasri and Nezamabadi-pour (2009) presented a new adaptive thresholding function for wavelet based noise removal. In their research, they introduced a new TNN combined with a new type of adaptive function with three shape tuning parameters to improve the Zhang's approach (Zhang, 2001). Golilarz et al. (2018) introduced a new method for hyperspectral remote sensing image de-noising utilizing 3D un-decimated wavelet transform with a new improved soft thresholding function to improve the results of previous threshold based noise removal. Qian (2018) proposed an algorithm for image de-noising utilizing an enhanced thresholding and median filter. One of the drawbacks and limitations of utilizing TNN based noise reduction is that it is time-consuming. Gradient-based learning is used in TNN to attain the optimum threshold value. Therefore, to address this problem, Bhandari et al. (2016) proposed optimized adaptive thresholding based image de-noising which they used JADE optimization algorithm instead of the steepest descent gradient based LMS method to decrease the computational time for attaining the optimum threshold value and other parameters.

To improve the efficiency of de-noising based on JADE algorithm, Golilarz et al. (2019b) utilized Harris Hawks optimization (HHO) algorithm (Heidari et al., 2019) in the first stage, and then improved adaptive generalized Gaussian distribution (AGGD) threshold function (Golilarz et al., 2019a) is used to enhance the quality of optimized based image de-noising approach, and lessen the computational time as well. The authors indicated that in improved AGGD based noise removal, the optimum value can be acquired without using any LMS learning and optimization algorithm. This advantage can save the processing time. In addition, the performance of wavelet thresholding can be enhanced using the adaptive GGD function because it provides us with more information about the image which the noisy constituents can be controlled well utilizing this adaptive function.

In this research, in the first stage we present adaptive soft and adaptive hard threshold to improve the results of standard hard and standard soft threshold function. In standard hard threshold, the small components are set to zero but adaptive soft and adaptive hard threshold can tune these coefficients using AGGD function in the interval [−σ_*n*_, σ_*n*_]. The results proved that adaptive threshold acts better than standard threshold in image de-noising. Additionally, to enhance the performance of image de-noising using optimization algorithms, improved adaptive generalized Gaussian distribution (AGGD) threshold is used for MRI brain image de-nosing. Moreover, we compared the proposed method with improved wavelet threshold proposed by Zhang et al. (2019). Experimental results prove the superiority of the proposed method over standard threshold, adaptive threshold, optimization (Golilarz et al., 2019b), and improved wavelet threshold (Zhang et al., 2019) based image de-noising methods.

## 2. Wavelet Based Image De-noising

To get the output de-noised image in the wavelet domain, we can do as follows (Golilarz et al., 2019b). Firstly, by applying wavelet transform we will get wavelet coefficients. These components can be sorted in two main groups: those carrying the most significant features of images and those having the non-important characteristics, with the former is the detail coefficient and latter is the non-important coefficients or noisy constituents. Next, these wavelet coefficients which we got from the first step, should be tuned using a suitable threshold value to preserve the crucial features and attribute of the image and discard the non-important components. These tuned components are called as thresholded wavelet coefficients. Then, it is time to apply the inverse wavelet transform (IWT) on these tuned thresholded wavelet coefficients providing us with the noise free image. On this matter, it is an important task to use a suitable threshold function and a threshold value since it plays an important role in getting our desired output de-noised image.

### 2.1. Definition of Noise, Threshold, and Mean Square Error

Assume that the noisy vector is as: $f={\left[f_{0},f_{1},\ldots .f_{N-1}\right]}^{T}$ which is contaminated by additive white Gaussian noise (AWGN):

(1) $$\begin{array}{l} f_{i}=u_{i}+n_{i},\;i=0,1,2,\ldots ,N-1 \end{array}$$

where, *u*_*i*_ represents the input noise-free wavelet constituents and *n*_*i*_ is the *iid* (independent and identically distributed) Gaussian noise.

Then, assume the data vector without noise as $U={\left[u_{0},u_{1},\ldots ,u_{N-1}\right]}^{T}$ and the thresholded output vector as $|Û={\left[{û}_{0},{û}_{1},\ldots ,{û}_{N-1}\right]}^{T}$ Admittedly, the main goal in image de-noising is to minimize the Mean Square Error risk (Nasri and Nezamabadi-pour, 2009). The Mean Square Error (MSE) risk can be obtained as follows:

(2) $$\begin{array}{l} mse_{risk}=\frac{1}{2}E{\left\|Û-U\right\|}^{2}=\frac{1}{2N}\overset{N-1}{\underset{i=0}{\sum }}{\left({\hat{u}}_{i}-u_{i}\right)}^{2} \end{array}$$

where, N is the size of the sub-band, (*u*_*i*_) is the input coefficients and ${\hat{u}}_{i}$ the thresholded wavelet coefficients (Nasri and Nezamabadi-pour, 2009).

Noise removal in the wavelet domain requires applying a proper threshold function and the threshold value. The universal threshold value (*t*_*uni*_) can be obtained based on VisuShrink technique using the equation below (Donoho and Johnstone, 1994). VisuShrink applies a universal threshold to all of the wavelet detail constituents. This threshold is known to discard additive Gaussian noise with high probability tending to result in overly smooth image appearance due to the fact that the threshold may be big because of its dependancy to the number of samples, *n*.

(3) $$\begin{array}{l} t_{uni}=\sigma \sqrt{2\ln\left(n\right)} \end{array}$$

where, *n* is the sample size and σ is the robust median estimator (Donoho and Johnstone, 1994) as follows:

(4) $$\begin{array}{l} \hat{\sigma }=Median\left(\left|G_{i,j}\right|\right)/0.6745 \end{array}$$

where, *G*_(*i, j*)_ is the components in the *HH*_1_ sub-band (Donoho and Johnstone, 1994).

### 2.2. Thresholding Neural Network (TNN)

**Figure 1:**
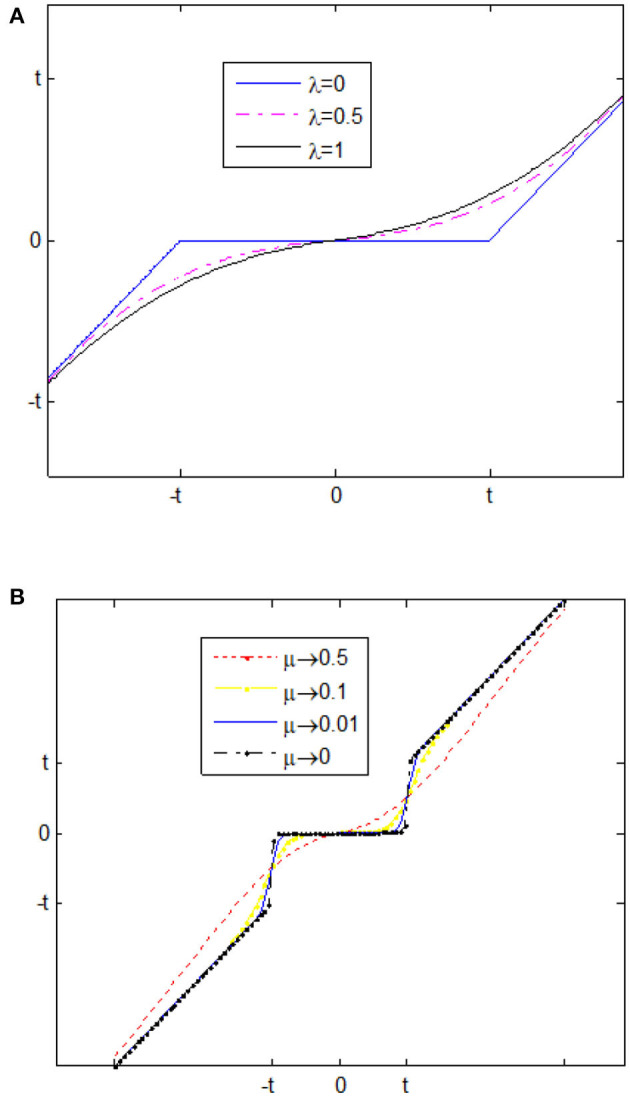
**(A)** is Zhang's improved soft threshold, and **(B)** is Zhang's improved hard threshold (Zhang, 2001).

Thresholding neural network based (space scale adaptive) noise reduction is proposed by Zhang (2001). In this network, there is linear transform which is fixed, and activation function which can be adaptive. The input of TNN is noisy image in which the linear orthogonal transform can be applied on it to get noisy components. Note that η is the non-linear activation function. The noisy coefficients need to be passed through this function to get thresholded wavelet coefficients. Eventually, by applying inverse linear orthogonal transform, we will attain output de-noised image (Golilarz and Demirel, 2018b). Zhang introduced two types of the non-linear threshold, namely: improved soft, and improved hard threshold functions as follows (Zhang, 2001). These functions with different λ and μ values are shown in Figure 1.

(5) $$\begin{array}{l} \eta_{soft}\left(x,t\right)=x+\frac{1}{2}\left(\sqrt{{\left(x-t\right)}^{2}+\lambda }-\sqrt{{\left(x+t\right)}^{2}+\lambda }\right) \end{array}$$

where, η_*soft*_ is the improved soft threshold function. Here, *x* is the wavelet component, *t* is the threshold value and λ > 0 is a user-defined function parameter (Zhang, 2001).

(6) $$\begin{array}{l} \eta_{\text{hard}}\left(x,t\right)=\left(\frac{1}{1+\exp\left(\frac{-x+t}{\mu }\right)}-\frac{1}{1+\exp\left(\frac{-x-t}{\mu }\right)}+1\right)x \end{array}$$

where, η_*hard*_ is the improved hard threshold function, *x* is the wavelet components, *t* is the threshold value and μ > 0 is a user-defined function parameter (Zhang, 2001). In this network, the optimum threshold value in the step *L* is given below (Golilarz and Demirel, 2017):

(7) $$\begin{array}{l} t\left(L+1\right)=t\left(L\right)-\Delta t\left(L\right). \end{array}$$

where, Δ*t*(*L*) is as:

(8) $$\begin{array}{l} \Delta t\left(L\right)=\theta \left(L\right)\frac{\partial J\left(t\right)}{\partial \left(t\right)},t=t\left(L\right) \end{array}$$

where θ is learning rate and *J*(*t*) is the MSE risk function.

To improve the efficiency and speed of Zhang's proposed TNN, Nasri and Nezamabadi-pour (2009) introduced a new thresholding neural network in the wavelet domain. Despite the Zhang's network which is space scale adaptive, this network is sub-band adaptive noise reduction (Nasri and Nezamabadi-pour, 2009). Similarly, the activation function is also non-linear and data-driven. The whole procedure of acquiring the de-noised image is mentioned above. In adaptive wavelet-based noise removal techniques, the threshold functions are chosen to be non-linear and adaptive. In this case, to improve the capability of the threshold functions, instead of setting the noisy components (below the threshold value) to zero by standard threshold functions, we can adjust and control these small coefficients using polynomial functions (Bhandari et al., 2016).

### 2.3. Optimized Based Image De-noising

Noise reduction using an optimized adaptive threshold function combined with nature-inspired optimization algorithms is introduced by Bhandari et al. (2016). The authors presented several optimization algorithms for satellite image de-noising. It was proved that image de-noising using TNN with steepest descent learning is time-consuming so that utilizing the optimization instead of LMS learning algorithm not only can improve the quality but also can increase the speed remarkably. Bhandari et al. (2016), utilized several different evolutionary for image de-noising. In their study, they used Differential Evolution (DE) (Storn and Price, 1997), Particle Swarm Optimization (PSO) (Poli et al., 2007), Wind Driven Optimization (WDO) (Bayraktar et al., 2011), Firefly Algorithm (FA) (Yang, 2010), Cuckoo Search (CS) Algorithm (Yang and Deb, 2009), and JADE algorithm (Zhang and Sanderson, 2009) as the optimizers to obtain the optimized thresholded wavelet coefficients in the process of getting the de-noised image. At the end, it was shown that using JADE algorithm performs better than other optimization algorithms in terms of PSNR values and qualitative results. By getting motivation from this paper, Golilarz et al. (2019b) attempted to improve the cited results by proposing a new technique. Then, it is proposed to apply another meta-heuristic optimizer (Harris Hawks Optimizer introduced by Heidari et al., 2019) instead of using JADE algorithm in the optimization process. The results showed the superiority of HHO based image de-noising method.

The main steps of obtaining the desired de-noised image using an optimization algorithm are as follows (Bhandari et al., 2016):

1. Apply a discrete wavelet transform on input noisy image (AWGN with zero mean and standard deviation of) to get noisy coefficients. Then, we can set the parameters of the optimization algorithm (number of iterations, number of solutions, scale parameters, etc.).
2. The noisy coefficients can be passed through an optimization algorithm consisting of the adaptive function so that the solution for the optimization algorithm can be acquired.
3. After computing it through threshold function, the best fitness values for each solution can be obtained (Bhandari et al., 2016).
4. After passing these parameters through adaptive function, we can get optimized thresholded wavelet coefficients.
5. Inverse discrete wavelet transform (IDWT) can be applied to these coefficients to get output de-noised image.

## 3. Adaptive Threshold for *t* = σ_*n*_

### 3.1. Adaptive Hard Threshold

**Figure 2:**
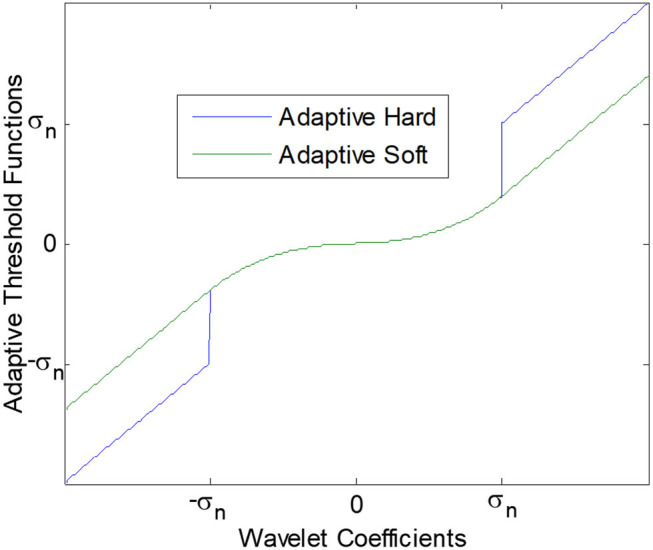
Adaptive threshold functions.

This function consists of two main parts: in the interval [−σ_*n*_, σ_*n*_], which is an AGGD oriented function, and behind the interval which is the identity function. As can be seen in Figure 2, since it is discontinuous, we call it an adaptive hard threshold function. We call this function as “tune and keep” since it keeps large coefficients behind the interval and unlike the standard hard threshold function, we can tune the small noisy coefficients instead of setting them to zero. This function is formulated below.

(9) $$\begin{array}{l} \chi \left(x\right)=\{\begin{array}{cc} x & ,x<-\sigma_{n}\\ s\left(x\right)-s\left(0\right), & |x|\leq \sigma_{n}\\ x & ,x>\sigma_{n} \end{array} \end{array}$$

where $s\left(x\right)=\sigma_{n}\left(e^{\frac{x^{2}}{2\sigma_{n}^{2}}-\frac{1}{2}}\right)$, *x* is the coefficient, and *t* = σ_*n*_ is the threshold value.

### 3.2. Adaptive Soft Threshold

The main difference between this function and the adaptive hard threshold is its continuity. As Figure 2 adaptive threshold function which is given below. We call this function as “tune and shrink” since it shrinks large coefficients behind the interval by the threshold value but unlike the standard soft threshold function, it is possible to tune the small noisy coefficients instead of setting them to zero.

(10) $$\begin{array}{l} \beta (x)=\{\begin{array}{ll} x+(t-s(t)-s(0))) & ,x<-\sigma_{n}\\ s(x)-s(0) & |x|\leq \sigma_{n}\\ x-(t-s(t)-s(0))) & ,x>\sigma_{n} \end{array} \end{array}$$

where, β(*x*) is the adaptive soft threshold, $s\left(x\right)=\sigma_{n}\left(e^{\frac{x^{2}}{2\sigma_{n}^{2}}-\frac{1}{2}}\right)$, *x* is the coefficient, and *t* = σ_*n*_ is the threshold value.

## 4. Improved AGGD Threshold for *t* > σ_*n*_

Golilarz et al., in 2019 proposed an adaptive generalized Gaussian distribution (AGGD) oriented threshold for image de-noising (Golilarz et al., 2019a). This function is data-driven, non-linear, and also flexible and fitted to any kind of images so that it can be shaped in various images. These are the most important characteristics of this function. It is proved that in the interval [−*t, t*], this function tunes the non-important constituents using an adaptive GGD threshold function instead of setting these coefficients to zero. Admittedly, this characteristic enhances the capability and flexibility of this function. The AGGD threshold function is given as (Golilarz et al., 2019a):

(11) $$\begin{array}{l} \rho \left(x\right)=\{\begin{array}{cc} x & ,x<-t\\ s\left(x\right)-s\left(0\right), & |x|\leq t\\ x & x>t \end{array} \end{array}$$

where, $s\left(x\right)=\sigma_{n}\left(e^{\frac{x^{2}}{2\sigma n^{2}}-\frac{1}{2}}\right)$, *x* is the coefficient, σ_*n*_ is the robust median estimator and *t* is the threshold value. This value is the inter section of x and *s*(*x*).

**Figure 3:**
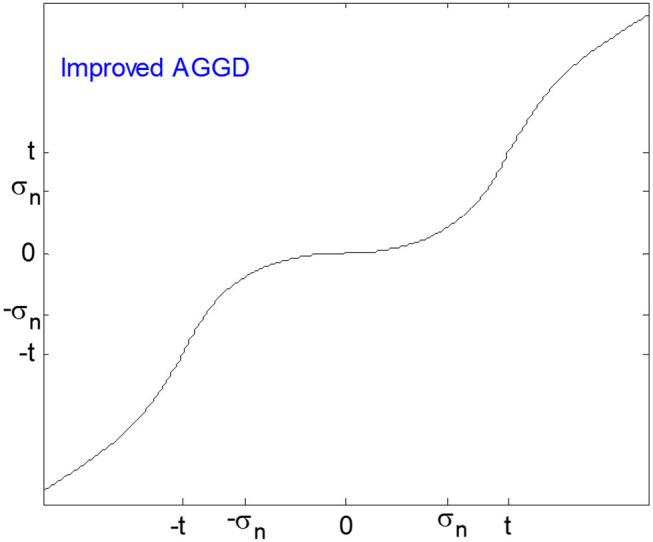
Improved AGGD threshold.

Golilarz et al. (2019b) improved the capability, quality, and speed of their former method (AGGD) by proposing an improved version of AGGD threshold function which results in an enhancement in both qualitative and quantitative results. This function is completely non-linear and differentiable by an adaptive generalized Gaussian distribution function in the interval [−t, t], and another non-linear function behind the interval. Obviously, the whole coefficients can be tuned using non-linear and data-driven functions. Like the AGGD threshold function, the threshold value can be obtained without using any optimization and steepest descent learning algorithms. Figure 3 shows improved AGGD function. This function is formulated as follows:

(12) $$\begin{array}{l} \mu \left(x\right)=\{\begin{array}{cc} \left(\frac{1}{1+e^{x+t}}\right)x-\frac{t}{2}, & x<-t\\ s\left(x\right)-s\left(0\right), & |x|\leq t\\ \left(\frac{1}{1+e^{-x+t}}\right)x+\frac{t}{2}, & x>t \end{array} \end{array}$$

where, μ(*x*) is the improved AGGD threshold, $s\left(x\right)=\sigma_{n}\left(e^{\frac{x^{2}}{2\sigma_{n}^{2}}-\frac{1}{2}}\right)$, *x* is the coefficient, σ_*n*_ is the robust median estimator and t is the threshold value.

## 5. Experimental Results

In this part we used four experiments to show the superiority of using improved AGGD method both qualitatively and quantitatively. In this research we used Peak Signal to Noise Ratio(PSNR) and MSE to evaluate the performance analysis of different de-noising techniques. MSE and PSNR (dB) can be obtained as follows:

(13) $$\begin{array}{l} MSE=\frac{1}{MN}\overset{M}{\underset{i=1}{\sum }}\overset{N}{\underset{j=1}{\sum }}{\left[d\left(i,j\right)-\hat{d}\left(i,j\right)\right]}^{2} \end{array}$$

where *d* is the original image, $\hat{d}$ is the de-noised image and *M*, *N* are the size of image (Bhandari et al., 2016).

(14) $$\begin{array}{l} PSNR=10{\text{log}}_{10}\left(\frac{255^{2}}{MSE}\right) \end{array}$$

where *MSE* is the mean square error.

In this part, we analyzed the use of wavelet based noise reduction with adaptive GGD threshold to improve the visual quality of MRI brain images in clinical researches and investigation which may be affected to unwanted noises during receiving and transmitting procedures. Particularly, we applied improved AGGD threshold on brain images in the wavelet domain to evaluate the effectiveness and efficiency of the proposed method in de-noising the medical images in comparison with other techniques.

**Figure 4:**
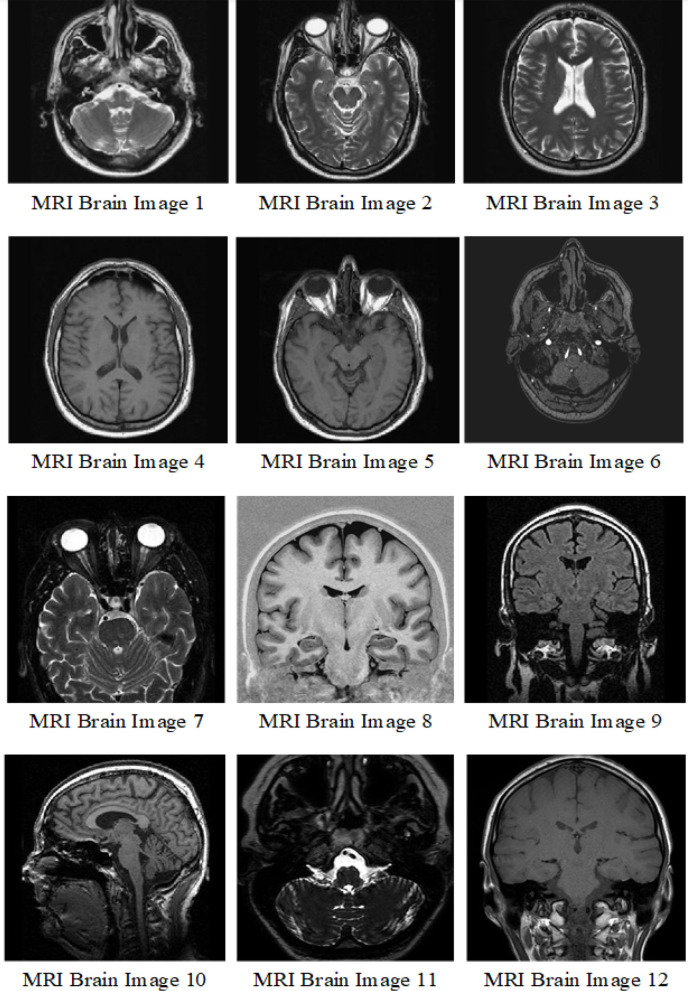
Original MRI brain images.

Here we utilized 12 MRI brain images which are shown in Figure 4. The dataset is available in Dataset (2020). The images are affected by additive white Gaussian noise AWGN with zero mean and different variance values. In these experiments we utilized Db4 wavelet with one level of decomposition. For HHO algorithm, the parameters are same with those in the original work of HHO (Heidari et al., 2019).

**Figure 5:**
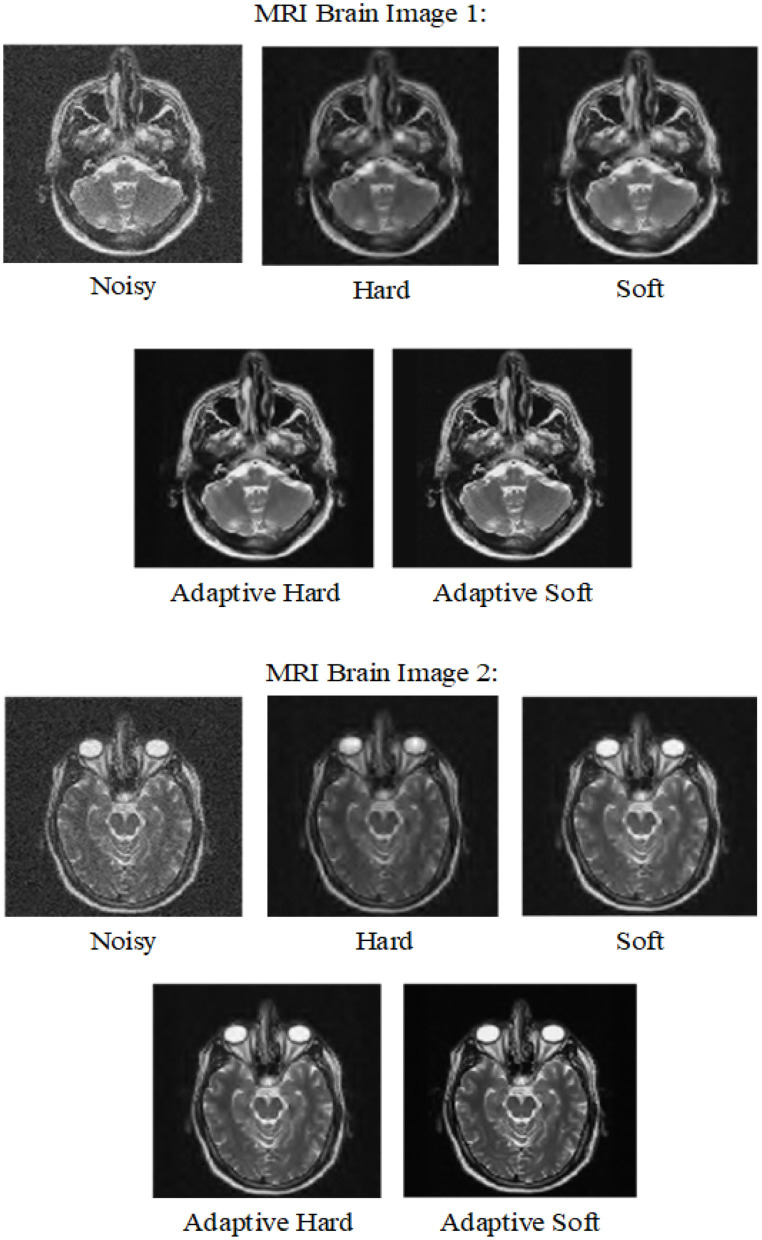
Visual comparison between adaptive and standard thresholds for variance 0.03.

In the first experiment, as can be seen from Tables 1, 2, we compare adaptive soft, adaptive hard with standard soft and standard hard threshold functions in terms of PSNR and MSE. In this experiment we used MRI images 1–2. In addition in Figure 5, we can see the visual comparison of these methods. It is obvious that adaptive soft threshold performs well comparing with adaptive hard, standard soft, and standard hard threshold function for image de-noising.

**Table 1:** Performance analysis of adaptive and standard threshold for MRI image de-noising in terms of PSNR values.

**Image**	**Variance**	**Hard**	**Soft**	**Adaptive hard**	**Adaptive soft**
MRI image 1	0.01	20.56	21.46	23.52	25.74
	0.03	19.39	20.41	21.83	23.80
	0.05	18.71	19.63	21.01	22.69
MRI image 2	0.01	22.78	23.47	25.61	27.39
	0.03	20.14	21.31	24.5	26.51
	0.05	19.56	20.7	23.81	24.83

**Table 2:** Performance analysis of adaptive and standard threshold for MRI image de-noising in terms of MSE.

**Image**	**Variance**	**Hard**	**Soft**	**Adaptive hard**	**Adaptive soft**
MRI image 1	0.01	571	465	289	173
	0.03	748	592	427	271
	0.05	875	708	515	350
MRI image 2	0.01	343	292	179	118
	0.03	630	481	231	145
	0.05	719	553	270	214

**Figure 6:**
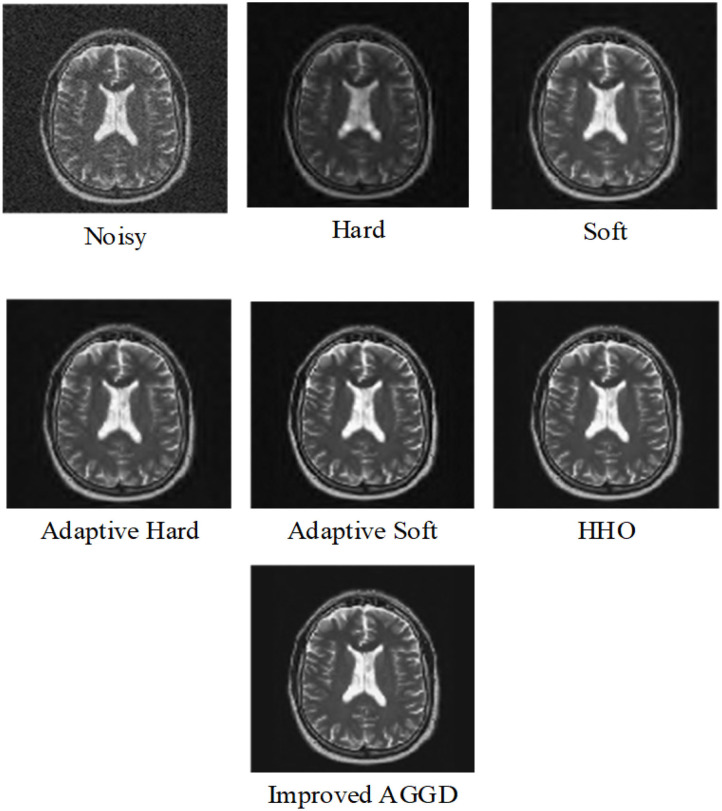
Comparison of visual inspection between different noise reduction methods for MRI Brain Image 3 for variance 0.03.

In the second experiment, in Tables 3, 4, we compared improved AGGD with de-noising using Harris Hawks Optimization (HHO) based noise reduction (Golilarz et al., 2019b), adaptive soft, adaptive hard, standard soft, and standard hard thresholds. Note that we used MRI Brain Image 3. Additionally, in Figure 6 we compared these techniques visually. It is obvious that improved AGGD performs better than other de-noising techniques.

**Table 3:** Comparison between different noise reduction methods in terms of PSNR values.

**Image**	**Variance**	**Hard**	**Soft**	**Adaptive hard**	**Adaptive soft**	**HHO**	**Improved AGGD**
MRI image 3	0.01	21.05	21.89	24.53	26.29	30.48	33.21
	0.03	19.69	20.43	22.48	24.46	29.11	32.14
	0.05	18.31	18.78	21.75	23.85	28.87	31.26

**Table 4:** Comparison between different noise reduction methods in terms of MSE.

**Image**	**Variance**	**Hard**	**Soft**	**Adaptive hard**	**Adaptive soft**	**HHO**	**Improved AGGD**
MRI image 3	0.01	510	421	229	153	58	31
	0.03	698	589	367	233	79	40
	0.05	959	861	434	268	84	49

**Figure 7:**
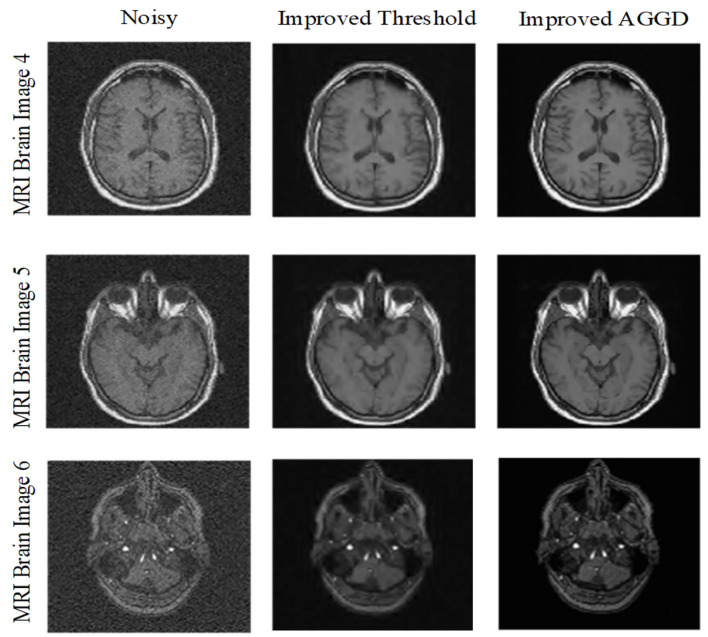
Visual comparison between improved AGGD and improved threshold for variance 0.03.

In the third experiment we compared proposed improved AGGD with improved threshold (Zhang et al., 2019). Here we used MRI Brain Images 4–6. As can be seen from Tables 5, 6, improved AGGD performs better than improved threshold function for MRI brain image de-noising. Moreover, Figure 7 shows the superiority of the proposed technique over improved threshold function qualitatively.

**Table 5:** Performance of improved AGGD compared with improved threshold in terms of PSNR values.

**MRI images**	**Variance**	**Improved threshold**	**Improved AGGD**
MRI image 4	0.01	27.68	33.39
	0.03	25.77	31.41
	0.05	24.35	30.36
MRI image 5	0.01	26.18	32.01
	0.03	25.73	30.76
	0.05	23.62	29.21
MRI image 6	0.01	27.07	32.79
	0.03	24.89	30.12
	0.05	23.51	29.03

**Table 6:** Performance of improved AGGD compared with improved threshold in terms of MSE.

**MRI images**	**Variance**	**Improved threshold**	**Improved AGGD**
MRI image 4	0.01	111	30
	0.03	172	50
	0.05	239	60
MRI image 5	0.01	157	41
	0.03	174	54
	0.05	282	78
MRI image 6	0.01	128	34
	0.03	210	63
	0.05	290	81

**Figure 8:**
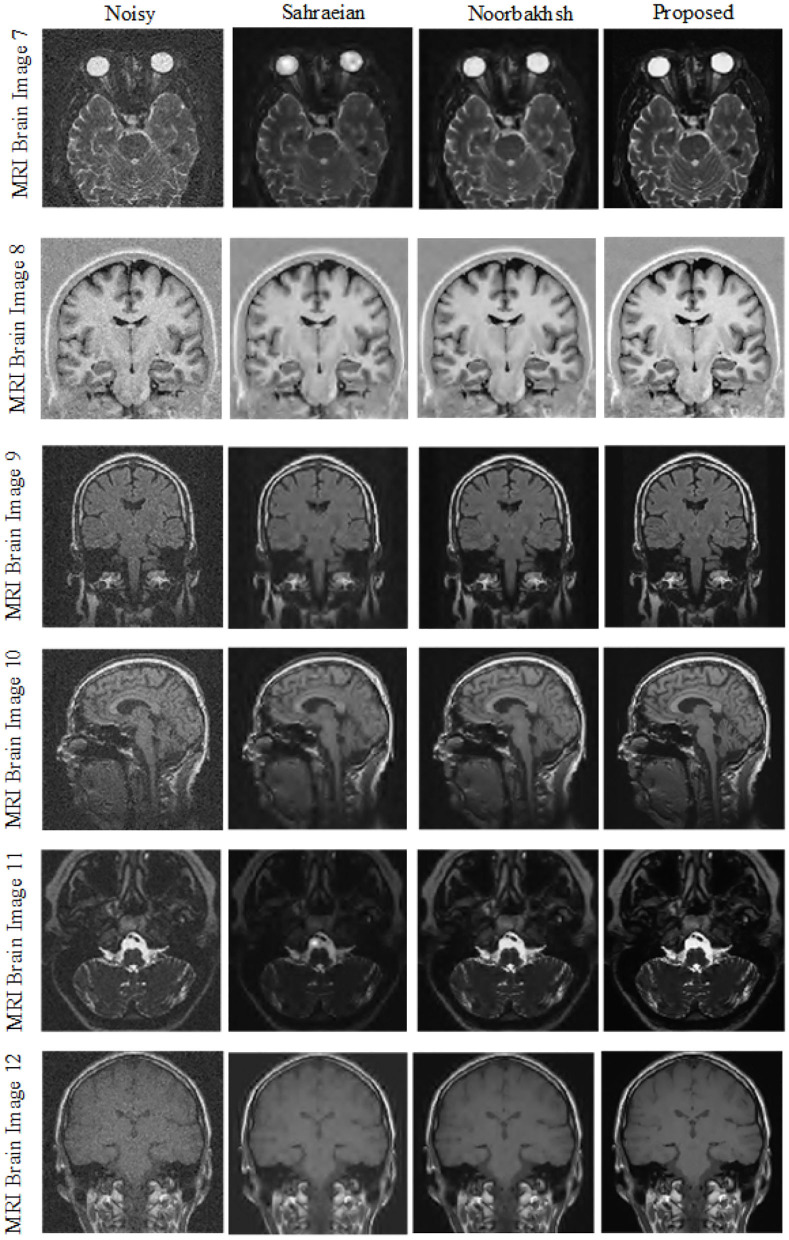
Visual comparison between different noise suppression techniques for variance 0.03.

In the fourth experiment we compared the proposed method with Sahraeian et al. (2007) and Noorbakhsh's technique (Golilarz et al., 2018) as well. Here we used MRI Brain Images 7–12. From Table 7 we can conclude that improved AGGD performs better than Sahraeian and Noorbakhsh's proposed method for MRI brain image de-noising. Additionally, we can see this comparison visually in Figure 8.

**Table 7:** Performance analysis of proposed method compared with Sahraeian and Noorbakhsh's technique for MRI brain image de-noising in terms of PSNR values.

**Image**	**Variance**	**Sahraeian**	**Noorbakhsh**	**Proposed**
MRI image 7	0.01	29.54	29.95	33.26
	0.03	28.25	28.64	32.07
	0.05	25.69	26.12	29.65
MRI image 8	0.01	29.78	30.21	33.31
	0.03	27.95	28.51	32.04
	0.05	25.76	26.11	29.51
MRI image 9	0.01	30.42	30.89	34.52
	0.03	29.16	29.63	33.01
	0.05	27.55	27.95	31.51
MRI image 10	0.01	30.39	30.76	33.77
	0.03	29.12	29.68	32.48
	0.05	26.38	26.91	30.1
MRI image 11	0.01	28.62	29.17	32.05
	0.03	27.43	27.87	31.01
	0.05	26.1	26.61	29.59
MRI image 12	0.01	30.03	30.56	33.46
	0.03	28.81	29.15	32.5
	0.05	27.42	27.98	30.89

The improved AGGD based image de-noising is presented to enhance both quality and the processing time. In the last experiment, we compared the processing time among various image de-noising methods. The computational cost of improved AGGD function is cheaper than improved threshold, HHO, adaptive soft, adaptive hard, standard soft and standard hard threshold functions. The speed and computational time among different noise suppression techniques has been compared in Table 8 for MRI brain Image 1 for variance 0.01. For HHO, the time is the average of 10 runs. For all the implementations and experimental results, we used Matlab programming language on a computer with Intel core i7 and 16 GB RAM.

**Table 8:** Processing time comparison among different techniques.

**Methods**	**Hard**	**Soft**	**Adaptive hard**	**Adaptive soft**	**HHO**	**Improved threshold**	**Improved AGGD**
Time (s)	4.2	4.6	3.5	3.2	4.4	2.6	1.8

## 6. Conclusion

In this study, a new method for wavelet-based MRI image de-noising is presented. Firstly, adaptive soft and hard threshold functions are introduced to improve the performance of standard threshold functions in the wavelet domain. Secondly, we used the newly emerged improved adaptive generalized Gaussian distributed oriented threshold function (improved AGGD) on the MRI images to show that, this data driven and image dependent threshold function performs well comparing with adaptive soft and hard threshold functions. Recently, image de-noising in the wavelet domain attracts lots of attentions in image and signal processing. Previous TNN and optimized based noise removal methods have good results but still the quality of an image needs to be enhanced and improved. TNN and optimized based noise reduction methods, require to utilize Least-mean-square (LMS) learning and optimization algorithms, respectively for acquiring the value of the optimum threshold and the parameters of the threshold functions which this process was time consuming. The improved AGGD based image de-noising is presented to solve these drawbacks. The computational cost of improved AGGD method is quite cheaper than the above mentioned techniques because we are in no use of LMS learning and optimization algorithms. This approach has good results in terms of PSNR values. The experimental analysis proves the superiority of improved AGGD threshold over adaptive threshold, standard threshold, improved wavelet threshold, and the optimized based noise reduction methods. For the future work, we will extend this work to deal with other forms of noise like impulse noise and non-Gaussian noise as well.

## Data Availability Statement

Publicly available datasets were analyzed in this study. This data can be found here: https://www.mr-tip.com/serv1.php?type=db1&dbs=Brain%20MRI.

## Author Contributions

NA, HG, RK, LA, YF, and CL conceived and designed the research and wrote the paper. HG, LA, YF, and CL provided the data. HG supervised the study. NA, LA, YF, and CL analyzed the data. HG, RK, LA, YF, and CL revised the manuscript. All authors contributed to the article and approved the submitted version.

## Conflict of Interest

CL was employed by The 54th Research Institute of China Electronics Technology Group Corporation, Shijiazhuang, China. The remaining authors declare that the research was conducted in the absence of any commercial or financial relationships that could be construed as a potential conflict of interest.
